# Functional Classification of Genome-Scale Metabolic Networks

**DOI:** 10.1155/2009/570456

**Published:** 2009-01-19

**Authors:** Oliver Ebenhöh, Thomas Handorf

**Affiliations:** 1 Max-Planck-Institute for Molecular Plant Physiology, Systems Biology and Mathematical Modeling Group, 14476 Potsdam-Golm, Germany; 2 Institute for Biochemistry and Biology, University of Potsdam, 14469 Potsdam, Germany; 3 Institute for Biology, Humboldt University, 10115 Berlin, Germany

## Abstract

We propose two strategies to characterize organisms with respect to their metabolic capabilities. The first, investigative, strategy describes metabolic networks in terms of their capability to utilize different carbon sources, resulting in the concept of *carbon utilization spectra*. In the second, predictive, approach minimal nutrient combinations are predicted from the structure of the metabolic networks, resulting in a characteristic *nutrient profile*. Both strategies allow for a quantification of functional properties of metabolic networks, allowing to identify groups of organisms with similar functions. We investigate whether the functional description reflects the typical environments of the corresponding organisms by dividing all species into disjoint groups based on whether they are aerotolerant and/or photosynthetic. Despite differences in the underlying concepts, both measures display some common features. Closely related organisms often display a similar functional behavior and in both cases the functional measures appear to correlate with the considered classes of environments. Carbon utilization spectra and nutrient profiles are complementary approaches toward a functional classification of organism-wide metabolic networks. Both approaches contain different information and thus yield different clusterings, which are both different from the classical taxonomy of organisms. Our results indicate that a sophisticated combination of our approaches will allow for a quantitative description reflecting the lifestyles of organisms.

## 1. Introduction

Genome-scale metabolic networks ideally comprise all enzymatic reactions that occur inside the cells of a specific organism. With the ever increasing number of fully sequenced genomes (at present, over 700 genome sequences have been published and well over 2000 sequencing projects are ongoing, [1]) and the advent of biochemical databases such as KEGG [2] or MetaCyc [3] in which the knowledge about the enzymes encoded in the genomes is compactly stored, organism-wide metabolic networks have now become easily accessible for a considerable number of species.

Whereas such models usually contain quite accurate information on the stoichiometry, that is the wiring, of the network, detailed knowledge on the kinetic properties of the enzymes catalyzing the involved reactions is still sparse. In the recent years, a number of analysis techniques have emerged which account for this fact and require only information about the stoichiometries of the participating reactions. A particularly useful framework is that of flux balance analysis which allows to infer optimal flux distributions given the structure of the network and an output function which is to be optimized. For the network of *E. coli*, for example, this approach has successfully been applied to predict flux distributions under the premise that biomass accumulation is maximized [4]. Further, in many cases, flux distributions could successfully be predicted for knock-out mutants lacking a particular enzyme [5].

In the recent past, we have proposed a complementary strategy for the analysis of large-scale metabolic networks, the so-called method of network expansion [6]. In this approach, networks of increasing size are constructed starting from an initial set of substrates (the seed) by stepwise adding all those reactions from the analyzed metabolic network, which use as substrates only compounds present in the seed or provided as products by reactions incorporated in earlier steps. The set of metabolites contained in the final network is called the scope of the seed and comprises all those metabolites which the network is capable of producing when only the seed compounds are initially available. Scopes can be understood as functional modules of the network, and since their compositions depend on the underlying network structure, they link in a natural way structural to functional properties of metabolic networks. In Ebenhöh et al. [7], we have systematically compared one particular metabolic function, namely, the ability to incorporate glucose as sole carbon source into the cellular metabolism, across species.

In this paper, we generalize these ideas and define for a large number of available genome-scale metabolic networks their *carbon utilization spectra*. Each spectrum characterizes the ability of a network to utilize different carbon sources. Groups of organisms with similar and different carbon utilization spectra are identified and compared with their evolutionary relatedness.

In Handorf et al. [8], we have studied the inverse scope problem and investigated whether it is possible to calculate from a given network structure a minimal set of seed compounds such that the corresponding scope contains a certain set of target metabolites. For the target, we have chosen important precursor molecules which are ubiquitous and essential for an organism's survival. By a systematic comparison of predicted nutrient requirements, we could identify global resource types and characterize each organism specific network by the degree of dependencies on each nutrient type. Here, we relate the two types of functional characterizations of organism-wide metabolic networks given by their nutrient profiles and their carbon utilization spectra, respectively. For this, we cluster organisms with similar predicted nutrient requirements and related carbon spectra and build phylogenetic trees based on the respective dissimilarities. This approach has been introduced in Aguilar et al. [9], where the so-called *phenetic* trees were constructed based on the reaction content present in the central metabolic pathways and compared to the classical 16S rRNA phylogeny. It was shown that within these phenetic trees, often those organisms are grouped which display a similar lifestyle, such as obligate parasitism. While these trees were constructed by comparing the *structure* of selected metabolic pathways, we attempt to build phylogenies based on *functional* properties of the complete organism-wide metabolic network. We generalize the ideas presented in Aguilar et al. [9] and outline how functional characterizations of networks may be put into relation with the particular lifestyles of the corresponding organisms. 

## 2. Carbon Utilization Spectra

For a given metabolic network, the scope of a particular combination of seed compounds defines what the network is in principle, by its stoichiometry, able to produce if exactly the seed compounds are available. By the inclusion of cofactor functionality (see methods for details), the interpretation of a scope as the biosynthetic capacity of an organism becomes realistic. An interesting question is how an organism may utilize a particular carbon source. We describe this capability using the concept of a scope by defining the seed as the set of all noncarbon-containing compounds appearing in the metabolic network of the organism under investigation. Additionally, we add to this set one particular carbon-containing metabolite. The scope of this seed describes the set of products that the organism is capable of producing when only the single carbon source is available but inorganic material is abundant. The description of an organism's metabolic capacity on a particular carbon source does not take into account whether this carbon source can actually be transported into the cell or only appears as an intermediate substrate of other biochemical processes.

For our analysis, we have retrieved 447 organism-specific metabolic networks from the KEGG database (see methods for details on the retrieval process). In order to characterize the ability to incorporate carbon sources, we have identified all metabolites which contain besides carbon only the chemical elements hydrogen and oxygen, resulting in a list of 935 simple carbon sources (the complete list is provided in Supplementary Material doi:10.1155/2009/570456). Applying the method of network expansion with the modification to allow for cofactor functionalities, we have calculated for each network and each carbon source the number of metabolites which can additionally be synthesized when only the carbon source and inorganic material are abundant. For a particular organism  and a specific carbon source , we denote this number by  and call it the biosynthetic capacity of the organism  on the carbon source . Interestingly, from 248 of the considered carbon sources, no organism is able to synthesize any new compounds. For these carbon sources,  for all organisms .

**Figure 1:**
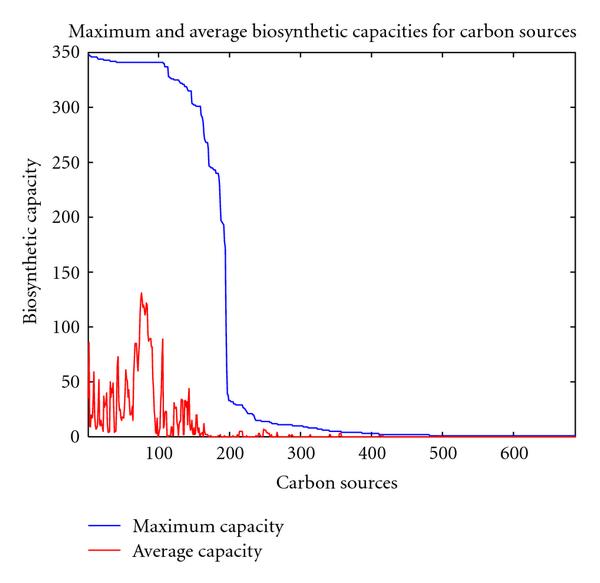
**Biosynthetic capacities for different carbon sources**. The blue line displays the maximum capacities found for an organism. The carbon sources are arranged along the -axis such that the maximal capacities appear in a decreasing order. The red line indicates the capacities for the carbon sources averaged over all considered 447 organisms.

In order to study how well different carbon-containing compounds may be metabolized by the various organisms, we characterize the remaining 687 carbon sources by two characteristic values. The maximum value of the biosynthetic capacities for organisms on a particular carbon source describes whether this carbon source is at all useful to at least one organism. The mean biosynthetic capacity when averaged over all organisms, on the other hand, describes the general utilizability of that carbon source. Figure 1 displays the maximal capacities for the various carbon sources. The carbon sources have been sorted by decreasing maximal capacity. Interestingly, the average capacity (red line) is not directly related to the maximal capacity. Apparently, while some carbon sources can be extremely well utilized by some specialized organisms, others can be utilized by a wider range of organisms. The highest biosynthetic capacity is observed for maltose. From this carbon source, *E. coli* may synthesize 348 new compounds. Also other common sugars, such as glucose, fructose, lactose, sucrose, or ribose, display a high maximal capacity in some organism. The highest biosynthetic capacity of a carbon source when averaged over all organisms is exhibited by pyruvate, from which on average 131 new metabolites may be produced. Remarkably, most metabolites occurring in the citric acid cycle, such as citrate, isocitrate, succinate, fumarate, malate, and oxaloacetate, also display a very high average biosynthetic potential, with over 110 compounds being producible from them by an average organism. This reflects the central role of these metabolites as precursor molecules for several amino acids and the pyrimidine nucleotide synthesis pathways. These metabolites give rise to the highest peak of the red curve in Figure 1. In contrast, from sugars, only fewer new compounds may on average be produced. For example, from glucose or maltose, the average organism may produce 86 new compounds and from sucrose only 62.

A sharp drop in maximal capacities can be observed, allowing to separate the carbon sources in two groups, a group displaying low capacities and a group of carbon sources for which there exists at least one organism that can utilize it to produce a considerable number of new products. In fact, for 491 carbon sources, there exists no organism able to produce more than 50 new compounds from it. The question arises whether simple chemical properties of the metabolites are responsible for this clear separation. Interestingly though, closely-related compounds may belong to different groups. For example, the - and -isoforms of arabinose exhibit maximal capacities of 341 and 2 compounds, respectively. This demonstrates that the separation and the biosynthetic capacity in general are not exclusively determined by chemical properties but rather reflect aspects of the biological roles of the metabolites. This finding is in agreement with our previous results obtained for the global metabolic network comprising all biochemical reactions found in the KEGG database [10].

**Figure 2:**
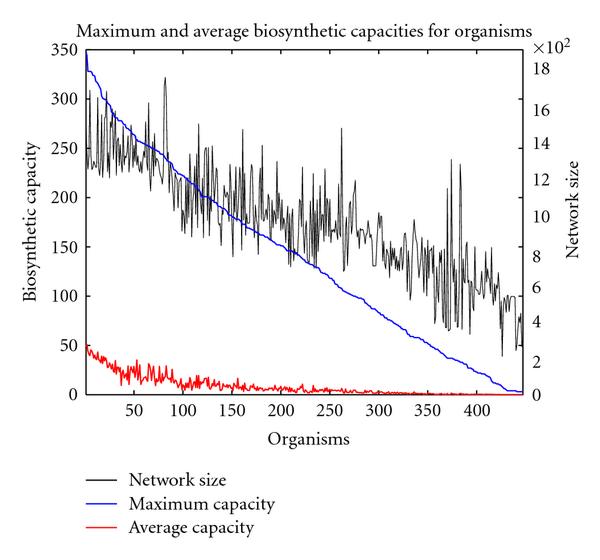
**Biosynthetic capacities for different organisms**. The blue line displays the maximal capacities. The organisms are arranged along the -axis such that the maximal capacities appear in a decreasing order. The red line indicates the normalized capacities for the carbon sources averaged over all considered 687 carbon sources. Additionally, the network size of the corresponding organisms is shown as a thin black line (right axis).

Analogous considerations can be performed for the different organisms. The maximal biosynthetic capacity is obtained from the carbon source that is ideally suited for a particular organism. On the other hand, the capacity averaged over all carbon sources characterizes the flexibility of an organism in terms of carbon usage. Figure 2 shows the biosynthetic capacities for all considered organisms. The blue line depicts the capacity an organism exhibited for the carbon source it may metabolize best. In analogy to Figure 1, the organisms are sorted such that the maximal capacity appears in a decreasing order. The decline of this curve is rather constant, in contrast to the maximal capacities for carbon sources. This implies that a separation of organisms into good and bad metabolizers is not easily possible, it rather appears that maximal capacities are approximately evenly distributed among the considered species. Interestingly, the capacity averaged over the carbon sources (depicted in red) shows a similar behavior as the maximal capacities, indicating that as a tendency organisms which can utilize a particular carbon source to produce a large number of new metabolites, can also efficiently use a number of alternative carbon sources. In fact, many strains of *E. coli* display both a high maximal capacity as well as a high average capacity (for strain K12 MG1655, the maximal and average capacities amount to 344 and 50.7, resp., for strain UTI89 348 and 48.8). This is not surprising since *E. coli* is a known generalist which can survive on many different carbon sources. Another interesting organism displaying a high maximal and average capacity (328 and 39.6, resp.) is *Rhodococcus sp. RHA1*, an organism with enormous catabolic potential that is able to live on contaminated soil [11]. An exception is *Vibrio fischeri* exhibiting a large maximal capacity by being able to produce 278 new metabolites from maltose, but a rather low average capacity of only 9.5 compounds. Interestingly, this bacterium is commonly undergoing symbiotic relationships with various marine animals such as bobtail squid, however, it may survive in isolation on decaying organic matter [12, 13].

The question arises whether the different capacities are simply a consequence of the network sizes, which may vary considerably among organisms. To test this, we have plotted in Figure 2 the number of metabolites within each organism-specific network as a thin black line. It can be observed that as a tendency the maximal capacity decreases with decreasing network size. However, the decrease in capacity is more pronounced, and the fluctuations in network size are relatively large, indicating that the network size is not the only determinant of the maximal capacity. The same finding is obtained when the numbers of reactions instead of the metabolites are used as a measure of network size (see Supplementary Figure S1).

**Figure 3:**
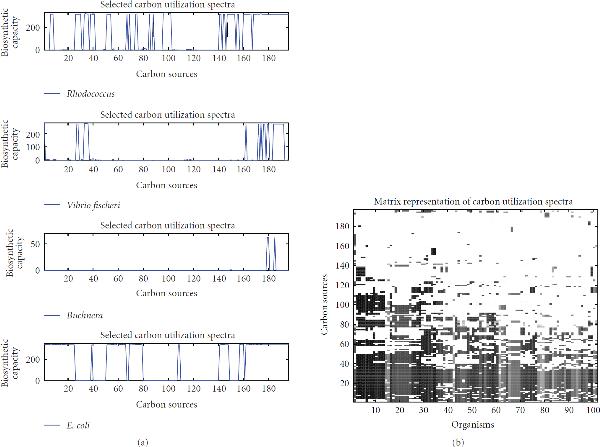
**Carbon utilization spectra**. (a) For the four selected species, *Rhodococcus, Vibrio fischeri, Buchnera,* and *E. coli* (from top to bottom), the carbon utilization spectra are explicitly plotted. (b) The carbon utilization spectra for a selection of 101 organisms are depicted in matrix form. Each column corresponds to an organism, while each row corresponds to one carbon source. Each spot indicates the biosynthetic capacity for a particular organism on a specific carbon source, with darker spots representing a higher capacity.

While the statistical properties of carbon usage of various organisms already allowed for some general statements, they are clearly insufficient to provide a detailed characteristics of an organism's ability to metabolize different carbon sources. For this, we introduce the concept of the *carbon utilization spectrum* of an organism. We define this spectrum as the set of biosynthetic capacities of the investigated organism for all usable carbon sources. In the following, we will focus on the 196 carbon sources that may be used by at least one organism to produce more than 50 new metabolites. A complete list of these carbon sources is provided in the supplementary material. For reasons of illustration and to demonstrate how spectra may be investigated and compared individually by visual inspection, we depict in Figure 3(a) the carbon utilization spectra for the four organisms: *Rhodococcus, V. fischeri, Buchnera,* and *E. coli*, which are all discussed in more detail throughout the paper. Each spectrum is a characteristic for a particular organism and describes which carbon sources the organism is able to incorporate into its metabolism. Clear differences between these spectra are directly visible. The generalist nature of *E. coli* and *Rhodococcus* is reflected by many large values; the high maximal but low average capacity of *V. fischeri* is manifested by a small number of high peaks. In contrast, *Buchnera*, an intracellular parasite, may only utilize a few selected carbon sources and possesses a small maximal capacity. In general, a comparison of different carbon utilization spectra allows the identification of commonly utilizable resources and those that are specific to single organisms.

A manual inspection is appropriate when focussing on a small number of organisms. For a large scale comparison of organisms as well as carbon sources, it is useful to simultaneously display all considered carbon spectra. This is performed as a matrix representation in Figure 3(b). Here, columns correspond to organisms and rows to carbon sources. The shading indicates the biosynthetic capacity for a particular organism using a certain carbon source, ranging from white (capacity of zero) to black, indicating the highest capacity amounting to 348 newly producible compounds. Therefore, each column represents a spectrum like the selected spectra depicted in Figure 3(a). For clarity, the representation is restricted to a selection of 101 organisms (the list is provided in the supplementary material). Further, the rows and columns of the matrix are arranged in such a manner that columns representing organisms with similar spectra are adjacent, and neighboring rows stand for carbon sources which may be used by a similar set of organisms. This matrix representation allows to easily identify universally usable carbon sources and those which can only be metabolized by a small group of organisms. The rows near the bottom of the graph as a tendency represent the universally usable sources, whereas those in the top half appear to be specific for the metabolism of only few organisms. Similarly, columns appearing on the left side of the graph as a tendency represent those organisms able to utilize a wide spectrum of carbon sources, while those near the right can only use a smaller set.

The selected spectra depicted in Figure 3(a) suggest that carbon sources either allow for the production of a large number of new metabolites or may not be metabolized at all. This assumption is also supported by the matrix representation in Figure 3(b). The vertical stripes result from the fact that within each row only extreme values are assumed. The capacity is either zero or close to the maximal capacity for that organism. Intermediate values are almost never observed. As a consequence, it is possible to divide the carbon sources for every organism in two groups, a group from which the organisms metabolism may produce a substantial amount of new substances and a group which it may not use for the production of other compounds. Inspired by this observation, we define for each organism  a *binary* carbon utilization spectrum represented by a binary vector  which is defined by (1)

The advantage of defining the spectra in a binary way is that the criterion whether a carbon source may be metabolized by a particular organism is independent from the actual number of new compounds that may be produced from it and also independent from other influencing factors such as the network size. Based on these independent spectra characterizing organisms by their ability to use different carbon sources, we define a dissimilarity measure which quantifies the different resource utilization capabilities of two organisms. Our dissimilarity measure is based on the Jaccard coefficient. This coefficient measures the similarity of two sets  and  by the ratio . It amounts to one for identical sets and to zero for completely disjoint sets. Let  and  denote two organisms and  and  their respective binary carbon utilization spectra. Converting the binary carbon utilization vectors  into sets , we introduce the distance measure (2)

For identical carbon utilization spectra, , whereas for disjoint spectra, .

**Figure 4:**
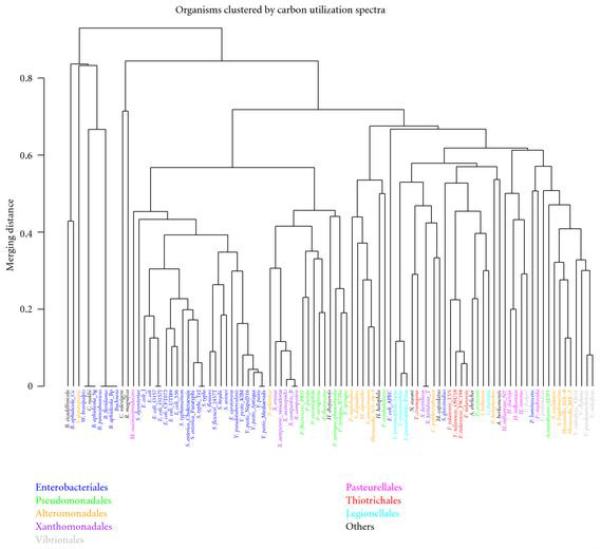
**Hierarchical clustering of all gamma-proteobacteria based on their binary carbon utilization spectra**. Families of gamma-proteobacteria have been color coded to indicate taxonomic similarities of the considered organisms.

We have applied these dissimilarities to perform a hierarchical clustering algorithm which clusters together those organisms exhibiting a similar carbon utilization spectrum. The resulting cluster dendrogram, restricted to the group of gamma-proteobacteria, is depicted in Figure 4. This figure demonstrates how this subgroup of organisms can in principle be grouped into clusters within which species exhibit similar carbon utilization spectra. Various families of gamma-proteobacteria are indicated with different colors. It can be seen that organisms belonging to the same family are often grouped together, indicating that they display similar carbon utilization spectra. However, for most families, exceptions can be found, demonstrating that taxonomically closely related organisms may exhibit drastically different carbon spectra.

All strains of *Yersinia pestis* are found in the vicinity of each other. Similarly, most strains of *Escherichia coli* are also located together. However, the strain *E. coli APEC*, which has been extracted from birds rather than humans, as is the case for all other *E. coli* strains included in our analysis, is grouped into a different cluster. This is surprising, since it was found in Johnson et al. [14] that this particular strain shares many traits with human uropathogenic *E. coli* strains (UTI89, 536, CFT073). Moreover, the authors showed a great sequence homology with 87–93% identity between these strains. These findings make it seem unlikely that the metabolism of *E. coli APEC* is so drastically different to other *E. coli* strains. Whether the differences in genomic sequence can really explain fundamentally different network functions or whether the available metabolic network of the *APEC* strain is simply under annotated remains to be investigated.

Clustering organisms by their carbon utilization spectra may reveal fundamental differences in the lifestyle of related organisms. For example, *Buchnera aphidicola*, an intracellular parasite in aphids [15], is evolutionary closely related to *E. coli*. However, whereas *E. coli* is widely known as a generalist that can survive in many different environments, *Buchnera* has adapted a specialized lifestyle strongly dependent on its host. The various strains of *Buchnera aphidicola* are grouped closely together with other bacteria that have specialized to a particular host; the most similar carbon utilization spectra are exhibited by the *Blochmannia* species *floridanus* [16] and *pennsylvanicus* [17], obligately intracellular bacteria in carpenter ants.

This detailed phylogenetic analysis demonstrates the usefulness of the concept of carbon utilization spectra. As expected, taxonomically related organisms often display similar spectra. However, since carbon utilization spectra characterize functional properties of metabolic networks, taxonomic closeness does not always result in similar carbon spectra. Rather, this new functional characterization allows to identify those particularly interesting cases in which similar and evolutionarily related organisms exhibit a different functional behavior.

It is an intriguing question whether organisms with similar carbon utilization spectra in general tend to inhabit similar environments. Since it is difficult to systematically characterize habitats and living environments, we have used two simple criteria to define four distinct classes of organisms. Firstly, we checked whether the enzymes catalase and superoxide dismutase are present in the organism's metabolism. With their ability to remove radical oxygen species, they are essential for survival in aerobic environments. Secondly, the ability to perform photosynthesis is characterized through the presence or absence of RuBisCO, the essential enzyme fixating one molecule of C to ribulose-1,5-bisphosphate to yield two molecules of phosphoglyceric acid. These classifications allow to define four categories of organisms with common lifestyle properties: organisms which are aerotolerant, potentially photosynthetic, none, or both.

**Figure 5:**
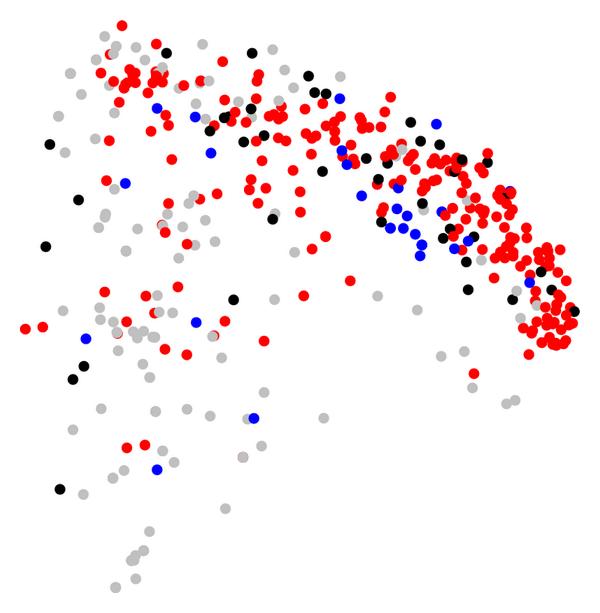
**Similarities of the carbon utilization spectra based on the Jaccard coefficient of the analyzed organisms are represented as a multidimensional scaling plot**. Red nodes denote aerotolerant organisms (catalase and super oxide dismutase enzymes present), while blue nodes mark organisms capable of carbon fixation (RuBisCO present). Organisms capable of both are black, while organisms capable of none are grey.

To study how carbon utilization spectra relate to these four categories, we have colored the organisms in Figure 4 according to the four categories (see Supplementary Figure S2). A visual inspection indicates that for organisms with common lifestyle properties, the tendency to be grouped together is comparable to the tendency observed for taxonomically related organisms. To test whether this observation also holds true when considering organisms from all kingdoms of life, we visualize dissimilarities in carbon utilization spectra as a two-dimensional scatter plot by applying multidimensional scaling [18]. The resulting scatter plot based on the distances (2) is shown in Figure 5. In this plot, every circle represents one organism, and those organisms are placed in close proximity, which exhibit similar carbon utilization spectra. The different categories are represented by different colors, with red circles characterizing aerotolerant organisms, blue circles potentially photosynthetic organisms. Species represented by black circles possess both properties, while species represented by grey circles possess none. A visual inspection hints at a nonrandom distribution of organisms sharing common lifestyle characteristics. The region near the top and the right of the figure contains a high concentration of aerotolerant organisms (red), and an agglomeration of potentially photosynthetic organisms (blue) is visible in the right half of the plane. To confirm this visual inspection, we have performed two statistical tests to demonstrate that the distribution of organisms within a particular class is indeed not random. First, we have compared the average distance  (2) between pairs of organisms within a class with the average distances calculated for a large ensemble of randomly selected subsets of organisms of the same size. If the classes indeed are clustered in particular regions of the graph, the observed average should be significantly lower than that observed in random subsets. However, it may still be possible that a class of organisms is concentrated in several regions that are far spread. To assess whether a class occupies locally concentrated regions, we have also tested whether small distances are over represented in the organism classes. For this, we have determined the fraction of distances between pairs of organisms within one class that is smaller than the 10% quantile of distances between all pairs of organisms. We again compared this number to that obtained for a large number of randomly selected subsets of organisms of the same size. For both, the potentially photosynthetic and the aerotolerant, organisms, less than 0.1% of randomly selected subsets of identical size displayed a smaller average distance or contained a larger fraction of small distances. The corresponding -values are indicated in Table 1.

**Table 1:** Statistics for distances calculated from the carbon utilization spectra (jaccard distance).

Ensemble (size)		-value		-value
RuBisCO (73)	0.687	.0006	0.159	.0008
SOD+CAT (279)	0.668	<.0001	0.158	<.0001
SOD+CAT+RuBisCO (41)	0.678	.0048	0.145	.0452

The ensembles of species belonging to common environmental categories are analyzed. The average distances and the fraction of small distances of the ensembles are compared to 10000 random sets of species of the same size as the corresponding ensembles. The expected value for the mean distance between two points is , and the expected value for the fraction of small distances by definition is . The -values were determined by comparing the distribution of the corresponding values for the random ensembles with the actually observed value for the selected ensembles. See Supplementary Figure S8 for more details.

This finding demonstrates that the defined lifestyle categories are not randomly distributed among all organisms and strongly indicates that the functional classification by carbon utilization spectra indeed reflects similarities of the habitats of organisms.

## 3. Nutrient Profiles

Using exclusively stoichiometric information on the metabolic networks of various organisms, we have in Handorf et al. [8] predicted minimal combinations of nutrients which an organism needs in order to produce all precursors that are required for essential life-sustaining processes such as the production of proteins, RNA or DNA, lipids, and important cofactors. As a result, for each organism, a nutritional profile has been predicted describing the essentiality of predefined resource types for the organism's metabolism.

Here, we compare these nutrient profiles of different organisms in order to obtain clusters of species possessing similar nutritional requirements. For this, the nutrient profile of an organism  is described as a vector . An entry  equals zero if nutrient type  is not needed, and equals one, if it is essential, and lies between these two extremes if the nutrient type represents one of several alternatives (the exact definition is given in the Methods). We define the dissimilarity between two organisms with respect to their predicted nutrient profiles by (3)

where the sum extends over all resource types.

Similarly to Figure 3, the nutrient profiles can be concisely represented as a matrix, which has been presented in Handorf et al. [8]. Also here, related organisms often possess similar nutrient profiles but exceptions exist. As also observed for the carbon utilization spectra, the closely related organisms *E. coli* and *Buchnera aphidicola* display significantly different nutrient profiles. In fact, the profile of *Buchnera aphidicola* predicts the essentiality of many nutrient types which are considered as typical for intracellular symbionts or parasites [8]. The profile of *E. coli*, on the other hand, shows only a few essential nutrients along with the possibility to use many alternative resources.

**Figure 6:**
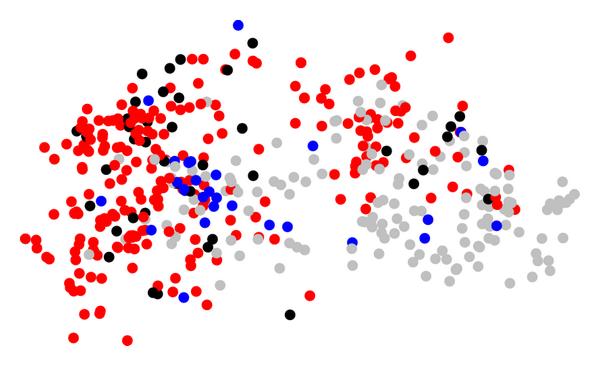
**Similarities of the nutrient profiles of the analyzed organisms are represented as a multidimensional scaling plot**. (Catalase and super oxide dismutase enzymes present), while blue nodes mark organisms capable of carbon fixation (RuBisCO present). Organisms capable of both are black, while organisms capable of none are grey.

In analogy to Figure 5, we perform a multidimensional scaling based on the distances  (3). The resulting two-dimensional scatter plot as shown in Figure 6. Again, each symbol represents one organism, and symbols with similar nutrient profiles are placed in close proximity. The color coding corresponds to that used in Figure 5.

The distribution of colors in Figure 6 is remarkable. As a tendency, identically colored symbols tend to concentrate in certain regions of the graph. For example, the left quarter seems dominated by aerotolerant organisms (red), and many potentially photosynthetic organisms (blue) seem to concentrate to the left of the center. However, also in this representation, the separation is not complete, and also closely neighbored nodes with different colors are abundant. To confirm our assumption that species within the same lifestyle category tend to be concentrated, we have again tested the mean distances within categories as well as the abundance of small distances against a large number of random selected subsets of identical sizes. We find that for both categories, the potentially photosynthetic and the aerotolerant organisms, none of 10000 randomly selected subsets of identical size displayed a smaller average distance or contained a larger fraction of small distances. The corresponding -values can be found in Table 2. These findings indicate that the clustering based on nutrient profiles is even more pronounced than that based on the carbon utilization spectra. We conclude that also the functional classification based on predicted nutrient profiles reflects aspects of typical habitats or the environments of the organisms.

**Table 2:** Statistics for distances calculated from the nutrient profiles.

Ensemble (size)		-value		-value
RuBisCO (73)	14.62	<.0001	0.185	<.0001
SOD+CAT (279)	14.57	<.0001	0.171	<.0001
SOD+CAT+RuBisCO (41)	14.52	.0011	0.218	.0002

The ensembles of species belonging to common environmental categories are analyzed. The average distances and the fraction of small distances of the ensembles are compared to 10000 random sets of species of the same size as the corresponding ensembles. The expected value for the mean distance between two points is , while the expected value for the fraction of small distances by definition is . As for Table 1, the -values were determined by comparing the distribution of the corresponding values for the random ensembles with the actually observed value for the selected ensembles. See Supplementary Figure S9 for more details.

## 4. Relating Network Structure, Function, and Phylogeny

We have provided two different measures to characterize organisms by functional aspects of genome-wide metabolic networks. Both methods seem suited to reflect differences and common properties of the typical habitats of the organisms. It is important to assess how far the information gained by the two approaches is independent and how the results were possibly influenced by structural the similarities of the organism's networks or by taxonomic proximity.

In the tree, we reconstructed from dissimilarities in carbon utilization spectra (see Figure 4), often pairs of closely related organisms were grouped together, however, also frequently related organisms were placed in different branches and it seemed that often parasitic organisms are grouped in close vicinity. This observation is in agreement with that of Aguilar et al. [9], where a similar tendency was observed when clustering organisms with respect to their reaction content of particular pathways. In both cases, the reconstructed tree does not reflect the standard taxonomy tree derived from rRNA sequence homologies. To assess the effect of the phylogenetic relationship and the purely structural properties of the networks, we have performed a topological comparison of four trees reconstructed from different dissimilarity measures. As a reference tree reflecting the commonly accepted evolutionary relationships between organisms, we have retrieved the taxonomy tree from the NCBI database [19] and extracted the minimal subtree containing all our considered 447 organisms as leaves. We have further constructed a tree by considering exclusively structural aspects of the metabolic networks by considering only their reaction content. However, in contrast to Aguilar et al. [9], we did not restrict this to single pathways or a small number thereof, but included all metabolic reactions present in the KEGG database. These two trees, in the following termed evolutionary and structural tree, were compared to the two functional trees, derived by hierarchical clustering based on the dissimilarity measures (2) and (3), the former trees reflecting differences in carbon utilization spectra, and the latter reflecting differences in nutrient profiles. Symmetric topological distances between these trees were calculated using the TREEDIST program of the PHYLIP [20] software suite, which is based on a tree metric introduced by Robinson and Foulds [21].

The symmetric tree distances are summarized in Table 3. Interestingly, the evolutionary tree is topologically more similar to the structural tree than to each of the functional trees. This indicates that phylogenetic proximity is stronger correlated with structural similarity than with common functional properties. This observation can be explained by considering that small alterations in the network structure may result in large functional changes.

**Table 3:** Comparison of tree topologies.

	Evolutionary tree	Structural tree	Carbon spectra	Nutrient profiles
Evolutionary tree	—	0.285	0.329	0.322
Structural tree		—	0.402	0.410
Carbon utilization spectra			—	0.439
Nutrient profiles				—

The tree distance was normalized with respect to the maximal possible values, such that identical trees exhibit a distance of 0, while maximally different trees have distance 1

Remarkably, when comparing the topologies of any of the functional trees with that of the structural tree, an even larger difference is observed. This also holds true when comparing both functional trees, derived from nutrient profiles and the carbon utilization spectra, respectively. This indicates that all three ways to describe metabolic networks contain fundamentally different pieces of information and that taxonomy, structure, and function of metabolic networks are only weakly correlated.

**Figure 7:**
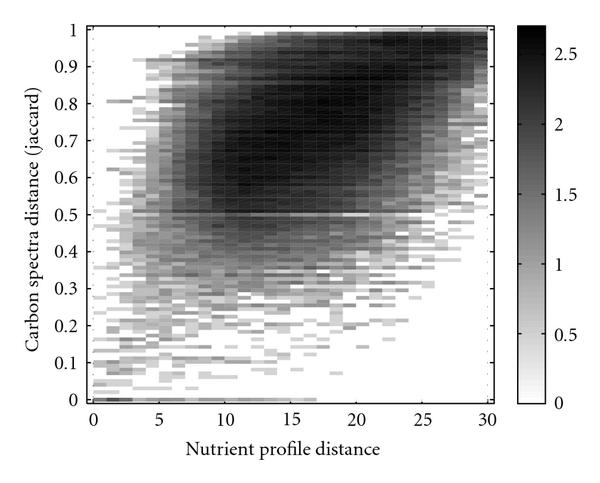
**Comparison of the distance measures obtained from the nutrient profiles and the carbon utilization spectra**. A two-dimensional histogram for all pairs of organisms is shown. Black shading indicates a high number of organism pairs sharing certain values of the two distance measures. The shading scale is logarithmic to allow for the visibility of relatively small abundances of combinations of distances.

Despite the differences manifested by the different tree topologies, the resulting functional classifications of the organisms nevertheless share common properties. We study how the distance measures are related by determining the number of organism pairs with a certain combination of dissimilarities. For  and , the corresponding numbers are plotted as a two-dimensional histogram in Figure 7, where dark spots indicate a high abundance of organism pairs. The scale for the intensity has been chosen logarithmically to make the smaller values visible. Considering that carbon utilization spectra strongly distinguish between similar chemical compounds but are restricted to single resources and a certain type of molecules, whereas nutrient profiles are of a more general nature, a strong correlation cannot be expected. However, because the global nutrient types also contain various carbon sources, these two measures are not completely independent, which is in agreement with the observed weak correlation.

Interestingly, organisms belonging to the same domain of life (archaea, eukaryota, and bacteria) also show a tendency toward clustering when multidimensional scaling is performed (see Supplementary Figures S3 and S4). However, the statistical significance is in general lower than for groups of organisms with common lifestyle properties (see Supplementary Tables S1 and S2). This observation is in agreement with our findings that dissimilarities based on carbon utilization spectra or nutrient profiles result in a different phylogeny when compared to the standard taxonomy as derived from the NCBI database.

To study how strong the functional distance measures (2) and (3) are correlated with the taxonomic proximity of organisms, we have defined a simple measure which crudely estimates the evolutionary distance. We denote this distance with  and define it by the number of edges that lie on the shortest path from organism  to organism  on the taxonomy tree derived from NCBI.

**Figure 8:**
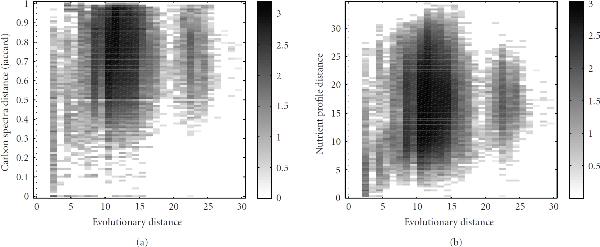
**Comparison of the functional distance measures obtained from (a) the carbon utilization spectra and (b) the nutrient profiles with the evolutionary distance**. A two-dimensional histogram for all pairs of organisms is shown. Black shading indicates a high number of organism pairs sharing certain values of the two distance measures. As in Figure 7, the intensity is scaled logarithmically since otherwise large regions would be invisible.

Figure 8 depicts two-dimensional histograms representing the correlation between the two functional distance measures  (Figure 8(a)) and  (Figure 8(b)) and the evolutionary distance . For both functional distances, no strong dependency on the evolutionary distance is visible. However, in particular for small evolutionary distances, the nutrient profiles are often similar, even though there exist exceptions as, for example, for the closely related species *E. coli* and *B. aphidicola* (see above). Also visible from Figure 8(b), species that have very similar nutrient profiles are often closely related. Similar observations can be made for the distances of carbon utilization spectra even though the correlation for small evolutionary distances is much less pronounced.

We have verified that also the tree based on predicted nutrient profiles differs strongly from the taxonomy tree (see Table 3). Remarkably, both functional trees as well as the structural tree are similarly distant from the taxonomy tree while exhibiting an even greater mutual distance. The fact that the distance measures are largely independent and that the structural and functional trees are topological very different shows that phylogenies built on sequences, network structures, or network functions contain independent information. We expect that a combination of structural and functional measures indeed allows for a reliable classification of organisms with respect to their habitat types.

## 5. Discussion

Based on purely structural information on the metabolic networks of a large collection of species, we provide two approaches to classify the organisms with respect to functional characteristics of their respective metabolism. For the first classification, the networks are probed with different carbon sources, and the variety of products that can be manufactured are calculated, leading to a functional characterization of the organisms by their carbon utilization spectra. In the second approach, minimal nutrient requirements are computationally predicted from the network structure, allowing for the characterization of organisms with respect to their nutrient profiles.

The characterization of organisms with respect to their biosynthetic capabilities from single carbon sources is useful to provide a characterization of both the organisms as well as the carbon sources. The presented considerations could clearly group the carbon sources into more and less utilizable. Similar to the dendrogram depicted in Figure 4, one can also group together the various carbon sources (see Supplementary Figure S5) to obtain information on their general usefulness. Carbon utilization spectra of organisms allow for a fine distinction for the usability of chemically similar organic compounds. However, the characterization only takes single carbon sources into account. It cannot be excluded that the metabolic networks of some organisms are structured in such a way that they cannot manufacture much from any single carbon source, but the combination of several will give rise to a high-biosynthetic potential. A disadvantage of the characterization of organism specific metabolic networks by their carbon utilization spectra is that they are not directly biologically interpretable. The ability to metabolize a carbon source is evaluated, regardless whether this carbon source is actually available in the environment or the organism possesses the necessary transporters to obtain this substance. We expect that with increasing knowledge on transport processes, it will be possible to adapt the concept of carbon utilization spectra to more realistically reflect the capacities of organisms in their respective environments. Despite the difficulties to directly relate carbon spectra with experimentally accessible quantities, we could show that by comparing carbon utilization spectra across a large number of organisms, distinct functional characterizations of metabolic networks can be obtained.

The second approach follows a more general strategy and tries to detect combinations from all chemicals which are sufficient for survival. Rather than simply describing biosynthetic capacities, minimal nutrient combinations are computationally predicted from the network structure. The predictive power of the inverse scope algorithm presented in Handorf et al. [8] is particularly pronounced for specialized compounds, such as vitamins, that an organism is not capable of producing and which, therefore, have to be supplied externally in the organisms diet. For nonessential nutrients, the predictions may be less accurate. For example, instead of sugars, the most important source for carbohydrates, the algorithm may predict other compounds from which sugars can, in principle, be produced, such as sugar phosphates or nucleotides. Indeed, while being a useful description of the strict requirements of organisms, nutrient profiles are not suitable to quantify the exploitability of specific substances since the algorithm considers only structural information and neglects kinetic details.

The two presented strategies to characterize organism-specific networks with respect to their metabolic functionality are, therefore, complementary, with nutrient profiles focussing on specialized nutritional components and carbon utilization spectra allowing to resolve the usability of chemically related carbohydrates. Despite the differing underlying concepts, both descriptions often lead to a similar characterization of closely related organisms, such as different strains of the same bacterial species. As a tendency this is expected, however, evolutionarily related organisms may have adapted to different environments. Both approaches are capable of reflecting differences in lifestyle for related organisms. For example, the generalist *E. coli* displays drastically different characteristics compared to its relative *B. aphidicus*, which, as a parasite in aphids, has evolved toward a high specialization and dependence on its constant environment.

This example demonstrates that the introduced functional measures, carbon utilization spectra and nutrient profiles, allow to distinguish between generalists and specialists. A future challenge will be to identify structural features of the metabolic networks that are responsible for an organism to be a specialist or generalist.

When comparing the phylogenetic trees derived from distance measures based on these two functional descriptions, we found that both trees differ considerably from the taxonomy tree, but differ even stronger when directly compared with each other. This observation supports our claim to have provided two complementary approaches, both yielding information not provided by the other approach.

By defining groups of strictly anaerobic and aerotolerant organisms as well as photosynthetic and nonphotosynthetic species, we could divide all organisms into four categories crudely characterizing their typical habitats. When clustering organisms with respect to their nutritional profiles, those organisms belonging to the same habitat type, are clearly not randomly distributed but appear to be concentrated in certain regions. It will be interesting to study how our proposed functional description relates organisms if a finer categorization of typical living environments is applied. We expect that it is possible to combine the two complementary functional categorization strategies to reliably cluster organisms with respect to typical habitats. Such a strategy would eventually allow for the prediction of the life-style of newly described and sequenced organisms and thus aid the discovery of suitable nutrient media and living conditions enabling a successful cultivation.

The presented concepts may also be used to view metabolism in an evolutionary context. With the proposed characterizations, the structural basis for functional changes which have occurred in the evolutionary history of organisms may be identified. For such studies, especially, pairs of organisms are interesting which are evolutionary closely related but show distinct functional characteristics. A problem remains to infer putative metabolic networks of common ancestral species. An approach how such networks may be estimated based on a maximum likelihood method is presented in Ebenhöh et al. [22], where we used the inferred networks to follow a particular metabolic function, the ability to incorporate glucose into the metabolism, along the evolutionary tree. A challenge for the future is to combine these two approaches and to arrive at a more detailed understanding of which specific structural properties of metabolic networks determine their functionality in different environments.

## 6. Methods

### 6.1. Network Retrieval

The analyses presented in this work are based on the same networks that we used in Handorf et al. [8] to infer nutritional requirements.

We have extracted the metabolic networks for 447 organisms from the KEGG database (as of Feb 13, 2007). The organisms have been selected in the following way. It has been verified that the number of reactions was realistic compared to similar organisms, if available. Otherwise, when the number of reactions seemed abnormally low, the original genome sequence paper was checked to verify that the low number is in line with biological knowledge, for example, in case of a low number of genes, a metabolic deficiency, and/or parasitism.

The corresponding metabolic networks have been extracted as follows. First, from the LIGAND subdivision (plain text file), the complete list of 6825 reactions has been imported. The reactions have been checked for consistency. We rejected 290 reactions because they showed an erroneous stoichiometry, by which we mean that some atomic species occurred in different numbers on both sides of the reaction. Further, we did not include 342 reactions involved in glycan synthesis because the focus of our investigation lies on the metabolism of small chemical species and does not include macromolecular syntheses.

Information on the reversibility of reactions has been extracted from the KGML files which specify the pathways for all organisms included in KEGG. In general, a particular reaction is listed in several KGML files, and the information on its reversibility may be ambiguous. In fact, we identified 136 reactions for which this is the case. For the present calculations, we consider a reaction to be irreversible only if it is defined as irreversible in all corresponding occurrences in the KGML files. This is the case for 2622 reactions.

The organism specific networks were determined using the "reaction" and "enzyme" files from the KEGG/LIGAND database. In a first step, for all reactions, the EC numbers of the catalyzing enzymes were retrieved from their corresponding entries in the "reaction" file (section ENZYME). Subsequently, from the "enzyme" file, for each enzyme, a list of organisms is obtained in which there exists a corresponding gene (section GENES). Thus, for each organism, the metabolic network is defined by all those reactions for which a catalyzing enzyme is encoded in its genome. In all cases where an enzyme is not fully classified (e.g., EC1.3.1.-), the corresponding entry in the "enzyme" file contains no GENES section. As a consequence, no such reactions are included in organism specific networks.

Further, the KO section of the database is inspected. Reactions specified in the DBLINKS/RN section of a KO entry are also assigned to the set of reactions of the organisms listed in the GENES section of this entry.

### 6.2. Network Expansion

The method of network expansion is a constructive strategy to identify all those compounds which can in principle be synthesized by a metabolic network when a defined set of substrates, the seed, is initially available. For this, networks of expanding size are constructed following the rule that in each generation, those enzymatic reactions from the metabolic network are added which use as substrates exclusively metabolites contained in the seed or which have been provided as products by reactions incorporated into the expanding network in previous generations. The expansion process stops when no further reactions may be added. The chemicals contained in the final network are called the scope of the seed and they describe what the network is in principle capable of producing from the seed metabolites.

To provide a realistic characterization of a biologically meaningful function, the concept of a scope in its original definition is too strict. In cellular metabolism, there exists a number of key compounds, the so-called cofactors, which participate in a large number of biochemical reactions in which they perform a characteristic function. For example, in many reactions, ATP acts as a donor of a phosphate group which is transferred to an acceptor molecule resulting in the formation of ADP. Similarly, NA may accept pairs of electrons, resulting in the release of NADH and thereby mediating redox reactions. Under physiological conditions, it is clearly unrealistic to assume that these cofactors need to be synthesized de novo from the available nutrients before they may act in their characteristic cofactor function. In Handorf and Ebenhöh [23], we have described the implementation of a modification of the expansion algorithm that takes this biological fact into account. We assume that cofactors may act in their typical functions even if they have not been synthesized from the available seed compounds in previous steps of the expansion algorithm. In Kruse and Ebenhöh [24], we have systematically compared the results obtained by network expansion with cofactor functionalities to that obtained by a mathematically more stringent approach based on flux balance analysis and found that network expansion provides an extremely good approximation while requiring orders of magnitudes less computation time.

### 6.3. Inferring Minimal Nutrient Combinations

To calculate a minimal combination of nutrient metabolites from which all precursor molecules necessary for higher level cellular processes may be synthesized, we have in Handorf et al. [8] described an algorithm that essentially reverses the scope algorithm. The greedy algorithm starts with a list of all metabolites occurring in the network. A seed containing this list is certainly sufficient to produce all precursor molecules. Then, the list is traversed, and each metabolite is temporarily removed. If after the removal of one metabolite still all precursors may be produced, this metabolite is permanently removed, otherwise it was required and is written back to the list. The list resulting after one complete traversal is minimal in the sense that no further metabolite may be removed without loosing the ability to produce all target metabolites. The resulting minimal combination strongly depends on the order in which the list is traversed. Therefore, we repeated this process a large number of times with perturbed lists. In order to obtain biologically meaningful combinations, we introduced heuristics that result in the preferential removal of large molecules from the list and the retaining of small molecules and those for which transporters are known. The resulting minimal combinations were then compared to identify exchangeable metabolites, and in this way, groups of metabolites could be identified from which at least one has to be provided as external resource. The cross-species comparison of such nutrient requirements led to the definition of global resource types, allowing for a quantification of the organism-specific requirements. For a particular organism  we define a vector  in which each component  is assigned the fraction of minimal nutrient combinations in which a representative of nutrient type  is found. Thus, an entry  characterizes the dependency of organism  on resource type  where a value of one indicates that resource type  is essential, zero signifies that the resource type is not required, and an intermediate value indicates that the resource type provides one of several alternatives.

### 6.4. Hierarchical Clustering and Dimensionality Reduction

The hierarchical clustering (see e.g., [25]) for Figure 4 was performed using the method hclust implemented in the software package R, using average agglomeration method. Dimensionality reduction for Figures 6 and 5 was obtained using two-dimensional scaling [18], implemented in R as method cmdscale.

### 6.5. Comparing Phylogenetic Trees

The tree comparison has been carried out using the treedist program from the phylip suite [20]. It calculates a distance between two trees by considering only its topology which was described by Robinson and Foulds [21]. The maximal distance of two trees with  species amounts to , yielding  for  species.

## Supplementary Material

Additional files in the gzip archive contain the complete lists of organisms and carbon sources as well as full matrices on the carbon utilization capacities.

## Supplementary Material

Supplementary Figures Tables Supplementary Figures: S1: As Figure 2 but with number of reactions as network size indicator, S2: As Figure 4 but with organisms colored to reflect environmental categories, S3, S4: Clustering of organisms, colors indicating the domains of life (archaea, bacteria, eukaryota), based on carbon utilization spectra (S3) and nutrient profiles (S4), S5: Cluster dendrogram of carbon sources, S6, S7: Cluster dendrogram of all 447 organisms, based on carbon utilization spectra (S6) and nutrient profiles (S7), S8: Statistics supporting Table 1, S9: Statistics supporting Table 2.
Supplementary Tables: S1, S2: As Tables 1 and 2 with additional information on the three domains of life. Click here for file

Full List Of Carbon Sources Contains the full list of all considered 935 carbon sources. Given are KEGG IDs and common names. Click here for file

All Organism List Contains the complete list of all 447 considered organisms. Given are the abbreviations used in KEGG and full names. Click here for file

Usable Carbon Sources A list of 196 carbon sources for which the maximal capacity exceeds 50. Click here for file

Full Carbon Utilization Matrix A space separated matrix with 935 rows and 447 columns. Entries give the number of comopunds that can be newly synthesized by an organism on that carbon source. Rows correspond to the carbon sources given in the file fullListOfCarbonSources.txt, columns to the organisms listed in the file allOrganismList.txt. Click here for file

Carbon Utilization Matrix Same as above, but restricted to the 196 carbon sources listed in the file usableCarbonSources.txt. Click here for file

Binary Utilization Matrix A binary utilization matrix containing 196 rows (corresponding to the carbon sources listed in usableCarbonSources.txt) and 447 columns (corresponding to the organisms listed in allOrganismList.txt). A non-zero entry indicates that a carbon source may be metabolized by an organism. Click here for file
