# Supplementary material for: Functional Classification of Genome-Scale Metabolic Networks
Source: EURASIP J Bioinform Syst Biol. 2009 Jan 19;2009(1):570456. doi: 10.1155/2009/570456 (PMC3171432; doi:10.1155/2009/570456)

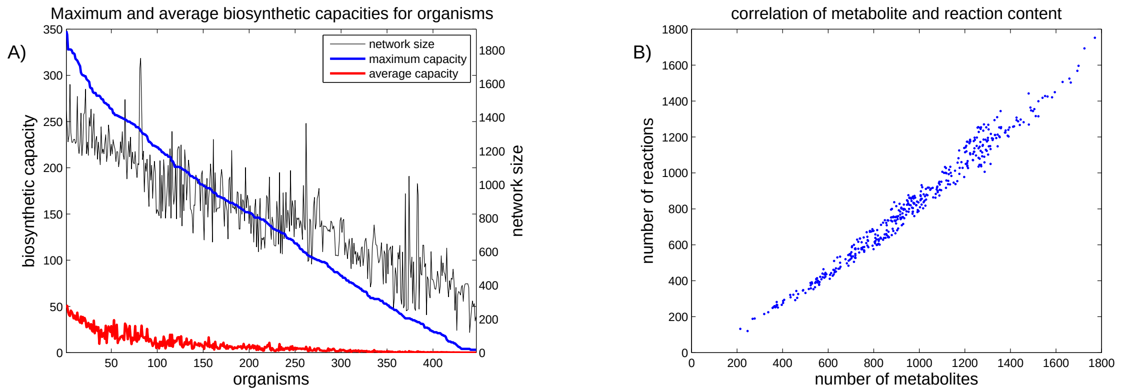

Figure S1: (A) Same as Fig. 2 in the main text but with the numbers of reactions used to indicate the network size instead of the numbers of metabolites. (B) Correlation between numbers of metabolites and numbers of reactions within all considered organism specific metabolic networks. The close similarity of Fig. S1A and Fig. 2 can be explained by the highly correlated metabolite and reaction content.

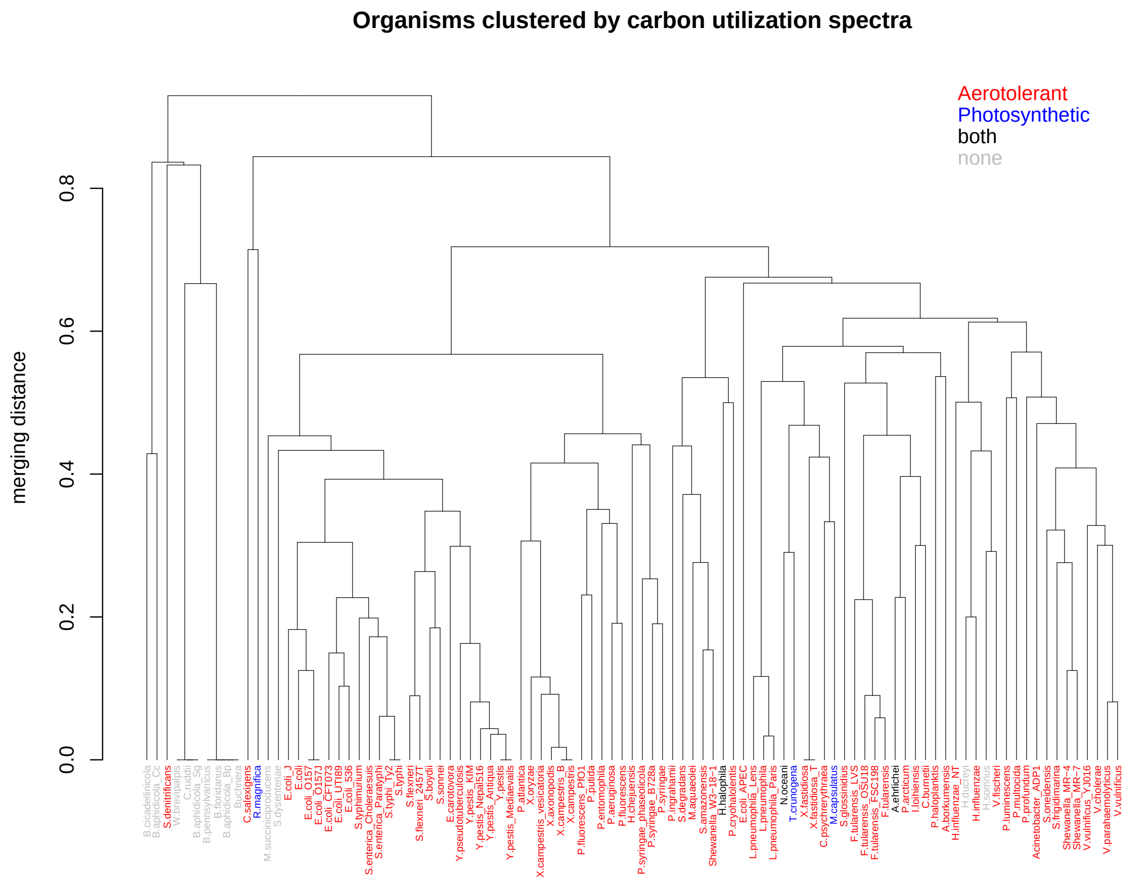

Figure S2: Same as Fig. 4 in the main text but organisms are color coded to represent the four lifestyle categories. Organisms in red are aerotolerant, organisms in blue potentially photosynthetic, organisms in black belong to both categories and organisms in grey to none of the two categories.

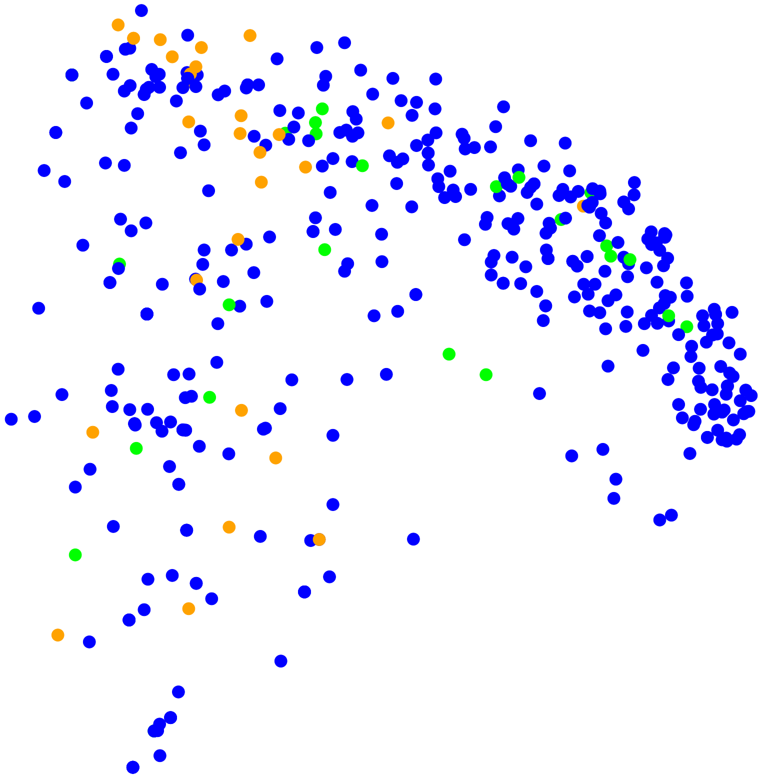

Figure S3: Multi-dimensional scaling plot based on distance d^J_{cus} with the three domains of life represented by different colors. Blue cirles represent bacteria, green circles eukaryota and yellow circles archaea.

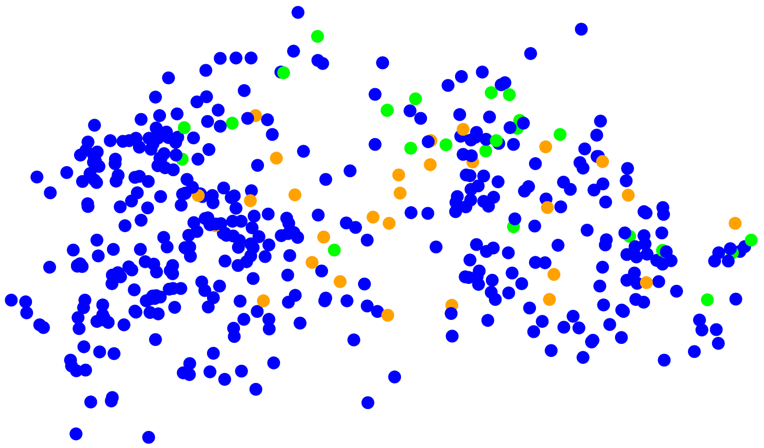

Figure S4: Multi-dimensional scaling plot based on distance d_{profile} with the three domains of life represented by different colors. Blue cirles represent bacteria, green circles eukaryota and yellow circles archaea.

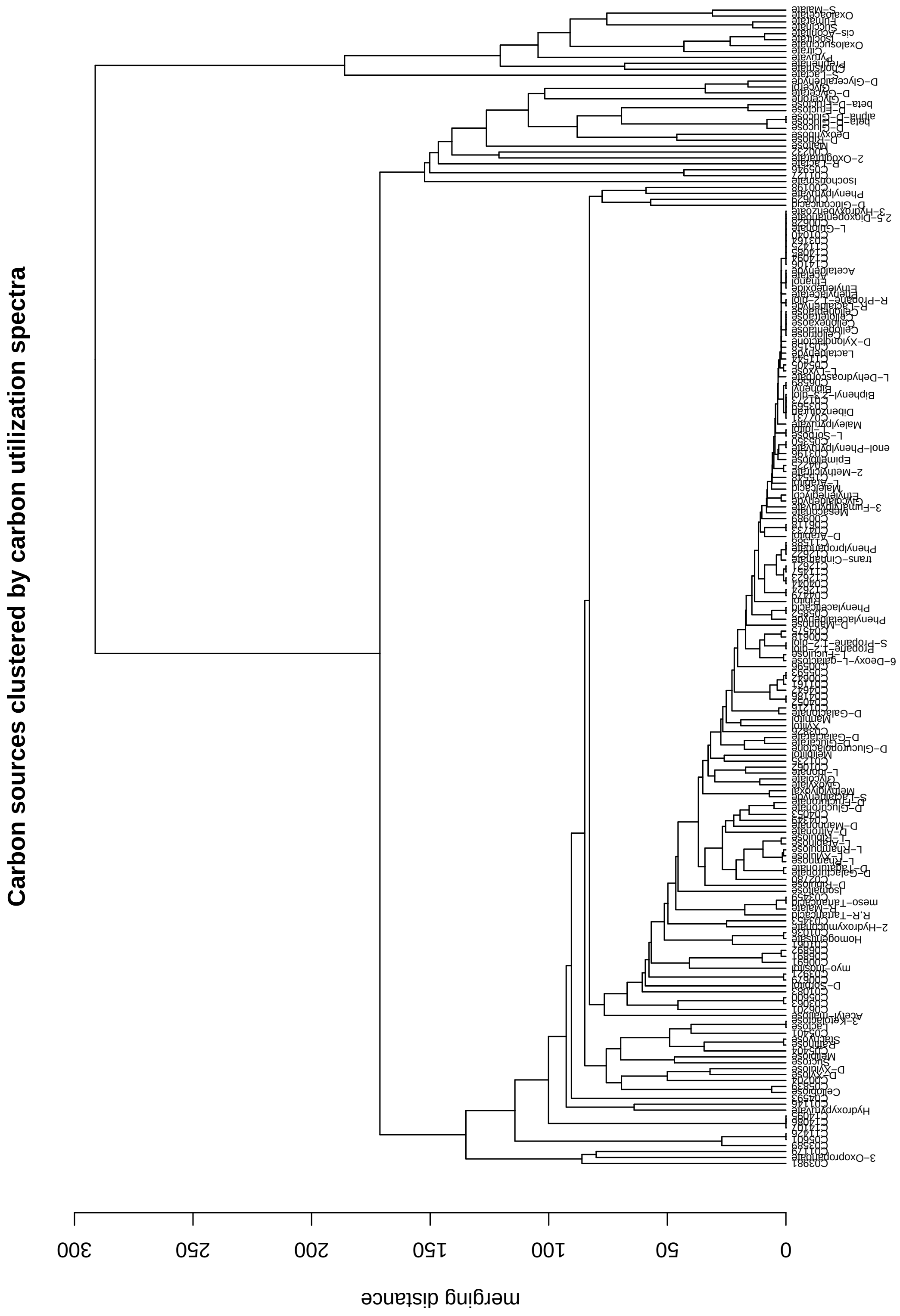

Figure S5: Cluster dendrogram for the carbon sources retrieved by hierarchical clustering. The distance is similar to d_{cus}, but the profiles were defined for carbon sources instead of organisms (corresponding to the rows in the matrix depicted in Fig. 3B).

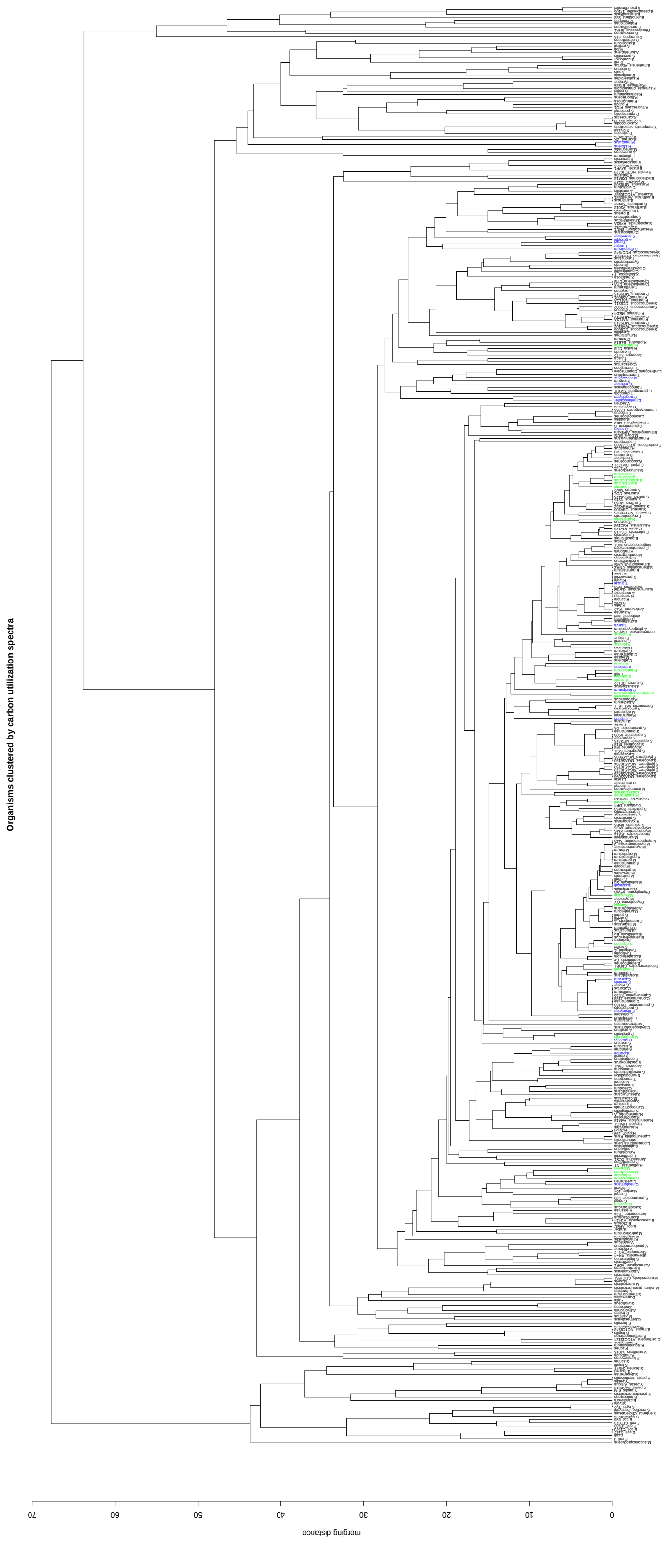

Figure S6: Cluster dendrogram including all 447 organisms retrieved by hierarchical clustering with the distances d_{cus}.

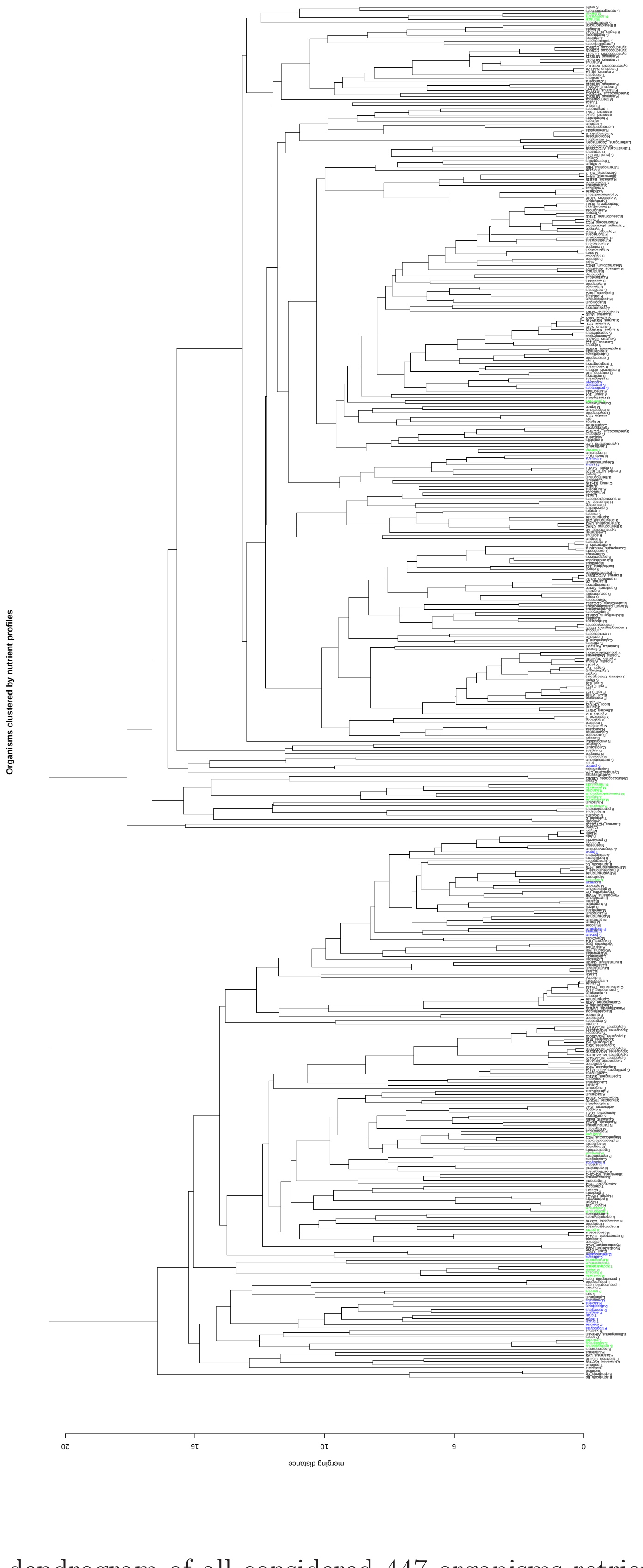

Figure S7: Cluster dendrogram of all considered 447 organisms retrieved by hierarchical clustering with the distances d_{profile}.

Table S1: Statistics for distances calculated from the carbon utilization spectra (jaccard distance). The expected value for the mean distance between two points is $E(\bar{d}) = 0.741$, the expected value for the fraction of small distances by definition $E(n_c) = 0.1$.

ensemble (size)	$\bar{d}$	p-value	n_c	p-value
RuBisCO (73)	0.687	0.0006	0.159	0.0008
SOD+CAT (279)	0.668	<0.0001	0.158	<0.0001
SOD+CAT+RuBisCO (41)	0.678	0.0048	0.145	0.0452
Bacteria (394)	0.738	0.1430	0.104	0.1314
Eukaryota (24)	0.719	0.2492	0.159	0.0574
Archaea (28)	0.646	0.0006	0.320	<0.0001

Figure S8: Distributions supporting table S1 (carbon utilization spectra): Shown are the distributions (red) for the average distance (1st row) and the fraction of small distances (2nd row) of the 10000 test sets for the categories RuBisCO, SOD+CAT, SOD+CAT+RuBisCO, Bacteria, Eukaryota and Archaea (left to right) as well as the corresponding value of the ensembles themselves (marked by vertical green line).

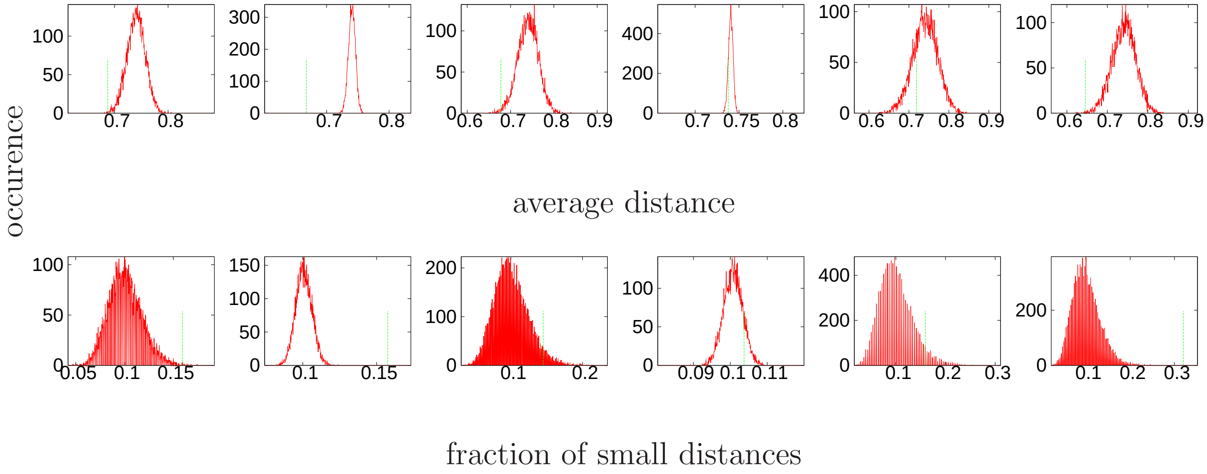

Table S2: Statistics for distances calculated from the nutrient profiles. The expected value for the mean distance between two points is $E(\bar{d}) = 16.56$, the expected value for the fraction of small distances by definition $E(n_c) = 0.1$.

ensemble (size)	$\bar{d}$	p-value	n_c	p-value
RuBisCO (73)	14.62	<0.0001	0.185	<0.0001
SOD+CAT (279)	14.57	<0.0001	0.171	<0.0001
SOD+CAT+RuBisCO (41)	14.52	0.0011	0.218	0.0002
Bacteria (394)	16.37	0.0011	0.114	<0.0001
Eukaryota (24)	13.92	0.0025	0.203	0.0136
Archaea (28)	14.82	0.0153	0.111	0.3480

Figure S9: Distributions supporting table S2 (nutrient profiles): Shown are the distributions (red) for the average distance (1st row) and the fraction of small distances (2nd row) of the 10000 test sets for the categories RuBisCO, SOD+CAT, SOD+CAT+RuBisCO, Bacteria, Eukaryota and Archaea (left to right) as well as the corresponding value of the ensembles themselves (marked by vertical green line).

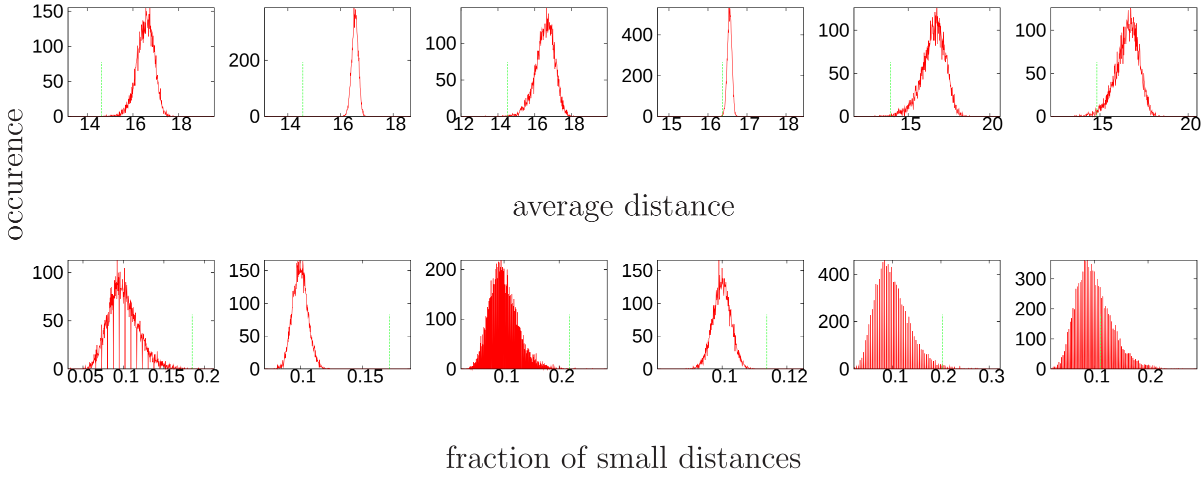

Supplement: Supplementary Figures Tables — Supplementary Figures: S1: As Figure 2 but with number of reactions as network size indicator, S2: As Figure 4 but with organisms colored to reflect environmental categories, S3, S4: Clustering of organisms, colors indicating the domains of life (archaea, bacteria, eukaryota), based on carbon utilization spectra (S3) and nutrient profiles (S4), S5: Cluster dendrogram of carbon sources, S6, S7: Cluster dendrogram of all 447 organisms, based on carbon utilization spectra (S6) and nutrient profiles (S7), S8: Statistics supporting Table 1, S9: Statistics supporting Table 2. Supplementary Tables: S1, S2: As Tables 1 and 2 with additional information on the three domains of life. [file 1687-4153-2009-570456-S1.pdf]
